# Alanine scan of sialorphin and its hybrids with opiorphin: synthesis, molecular modelling and effect on enkephalins degradation

**DOI:** 10.1007/s00726-018-2585-8

**Published:** 2018-05-12

**Authors:** Małgorzata Sobocińska, Artur Giełdoń, Jakub Fichna, Elżbieta Kamysz

**Affiliations:** 1 Laboratory of Chemistry of Biological Macromolecules, Department of Molecular Biotechnology, Faculty of Chemistry, University of Gdansk, Wita Stwosza 63, 80-308 Gdansk, Poland; 2 Laboratory of Simulation of Polymers, Department of Theoretical Chemistry, Faculty of Chemistry, University of Gdansk, Gdansk, Poland; 3 Department of Biochemistry, Faculty of Medicine, Medical University of Lodz, Lodz, Poland

**Keywords:** Enkephalins, Opiorphin, Sialorphin, Neutral endopeptidase, Peptides synthesis, Molecular modelling

## Abstract

Enkephalins are involved in a number of physiological processes. However, these peptides are quickly degraded by peptidases, e.g. the neutral endopeptidase (NEP). Inhibition of the enzymatic degradation of enkephalins is one of the possible approaches to prolong their activity. Selective inhibitor of NEP, sialorphin, is the attractive lead compound for enkephalins degradation studies. In this work, an alanine scan of sialorphin and a series of its hybrids with opiorphin, synthesised by the solid phase method, were performed. The effect of the peptides on degradation of Met-enkephalin by NEP in vitro was investigated. Molecular modelling technique was used to identify residues responsible for protein–ligand interactions. We showed that substitution of amino acids Gln^1^, Pro^4^ and Arg^5^ of sialorphin for Ala significantly reduced the half-life of Met-enkephalin in the presence of NEP. [Ala^3^]sialorphin displayed a higher inhibitory potency against NEP than sialorphin. Substitution of His^2^ for Ala led to a compound which was as active as lead compound. Sialorphin has a structure which hardly tolerates substitution in its sequence at positions 1, 4 and 5. The conversion of His^2^ for alanine in sialorphin is tolerated very well. The higher inhibitory potency of [Ala^3^]sialorphin than sialorphin against NEP is caused by removal of the hydrophilic residue (Asn) and a better fit of the peptide to the enzyme-binding pocket. The role of side chains of sialorphin in degradation of enkephalin by NEP has been explored. This study also provides an important SAR information essential for further drug design.

## Introduction

Enkephalins, including Met-enkephalin (Tyr–Gly–Gly–Phe–Met) and Leu-enkephalin (Tyr–Gly–Gly–Phe–Leu) are involved in the pain modulating mechanism in the spinal cord (Hughes et al. 1975; Noble et al. 1992) and in the immune modulating system (Hiddinga et al. 1994; Marksteiner et al. 1993; Stefano et al. 1998). They have beneficial impact on the function of the digestive system and exert anti-inflammatory effect through direct influence on the immune cells (Owczarek et al. 2011). However, due to high susceptibility to degradation by a group of enzymes termed enkephalinases such as neutral endopeptidase (NEP; EC3.4.21.11), aminopeptidase N (APN; EC3.4.11.2), angiotensin-converting enzyme (ACE; EC 3.4.15.1), and dipeptidyl peptidase III (DPP III; EC 3.4.14.4) and the resulting short half-life in vivo, the therapeutic potential of enkephalins is relatively low. Inhibition of enkephalins degradation by enzymes is one of the possible approaches to prolong their activity. Opiorphin and sialorphin are NEP inhibitors that lengthen the half-life of enkephalins in vitro (Kamysz et al. 2016).

Opiorphin (Gln–Arg–Phe–Ser–Arg) is a human endogenous pentapeptide that was first detected in human saliva, encoded by human PROL1 gene (Wisner et al. 2006). It is a dual physiological inhibitor of NEP and APN (Wisner et al. 2006; Thanawala et al. 2008). Its functional homologue, pentapeptide sialorphin (Gln–His–Asn–Pro–Arg), was found in submandibular glands and prostate of rats, encoded by rat Vcsa1 gene. It is a selective inhibitor of NEP (Rougeot et al. 2003; Messaoudi et al. 2004; Davies et al. 2007). Intensive research on the expected pharmacological effect of opiorphin and sialorphin revealed their dual action, resulting from both the increase in enkephalins level via blocking degradation enzymes, as well as a direct interaction with the binding sites. Endogenous enkephalin-degrading enzyme inhibitors were shown to affect various pathways in the mammalian body. For instance, opiorphin displays analgesic and antidepressant activity in animal models of pain and depression (Wisner et al. 2006; Popik et al. 2010). The compound is also active in vivo, showing a pain-suppressive potency similar to morphine in chemical and mechanical rat models of pain (Wisner et al. 2006; Tian et al. 2009; Rougeot et al. 2010). Noteworthy, these actions were not accompanied by side effects typical of opioids, such as development of tolerance or addiction (Popik et al. 2010; Rougeot et al. 2010). Sialorphin is released locally and systemically in response to stress (Rougeot et al. 2003). It displays a potent, naloxone-sensitive antinociceptive effect in behavioural models of acute pain, plays an important role in the control of social behaviour, enhances sexual behaviour and erectile function in male rats (Messaoudi et al. 2004). As reported recently, sialorphin attenuated acute, semichronic as well as relapsing 2,4,6-trinitrobenzenesulfonic acid (TNBS)-induced colitis in mice after systemic administration, its anti-inflammatory action being associated with the mu and kappa opioid receptors (Kamysz et al. 2016; Salaga et al. 2017).

The purpose of this work was to explain the role played by the residues located at the specific positions in the sialorphin and opiorphin by the alanine scan of sialorphin and a series of its hybrids with opiorphin. The peptides were characterised in vitro by the measurement of their effect on degradation of Met-enkephalin by NEP. In this study, we also used molecular modelling techniques to identify residues responsible for protein–ligand interactions of the inhibitors with NEP. Having all the identified contacts in hand, we could explain the influence of the inhibitor residues on the observed activity.

## Materials and methods

### Peptides synthesis

All the peptides were obtained manually by the solid phase method by stepwise coupling of Fmoc-amino acids to the growing peptide chain on a 2-chlorotrityl chloride resin (loading 0.3–0.9 mmol/g, 1% DVB, 200–400 mesh, Orpegen Peptide Chemicals GmbH, Heidelberg, Germany). *N*^α^-Fmoc-protected amino acids and reagents used for the solid phase synthesis were acquired from Iris Biotech GmbH (Marktredwitz, Germany). The amino acid side chain protecting groups were Trt for His, Gln and Asn and Pbf for Arg. First amino acid was bound to the resin according to Barlos et al. (1991) with a loading dose of 0.7 mmol/g. Peptide chains were elongated in consecutive cycles of deprotection and coupling. Deprotection was performed with a 25% piperidine solution in *N,N*-dimethylformamide (DMF), whereas the chain elongation was achieved with equimolar mixtures of a protected amino acid derivative (Fmoc-AA) dissolved in DMF with addition of 1-hydroxybenzotriazole (HOBt) and *N,N′*-diisopropylcarbodiimide (DIC) for 2 h. Three equivalents (based on the resin reactive groups) of these reagents were used. The efficiency of the coupling reactions was checked using the chloranil test (Vojkovsky 1995). The peptides were cleaved from the resin and the protecting groups were removed in one step using a mixture of trifluoroacetic acid (TFA):triisopropylsilane (TIS):H_2_O (95:2.5:2.5, v/v/v) and stirred for 2 h. The peptides were precipitated with ice-cold ether and lyophilised. Crude peptides were purified by reversed-phase high-performance liquid chromatography (RP-HPLC) on a Kromasil C8 column (8 mm × 250 mm, 100 Å pore size, 5 µm particle size), using two solvent systems of 0.1% TFA in water [A] and 0.1% TFA in acetonitrile [B] and a linear gradient of 2–60% B over 40 min (peptides III–VII), and 2–40% B over 30 min (peptides I, II, VIII and X–XI), and 2–25% B over 20 min (peptide IX), all at a flow rate of 10 mL/min and the eluent was monitored at 214 nm. The purity of the peptides was checked on a Beckman HPLC controlled by an Lp-Chrom system. Fractions containing the pure peptides (> 98%) were pooled and lyophilised. Matrix-assisted laser desorption/ionization mass spectrometry (a Biflex III MALDI-TOF instrument, Bruker Daltonics, Germany) was used to confirm identity of the pure products. Physicochemical properties of the peptides are shown in Table 1.

**Table 1:** Physicochemical properties of peptides I–XI

Peptide	Name	Molecular formula	HPLC *t*_R_ [min]	Molecular ion	
				Calc. [M]^+^	Found [M + H]^+^
I	Sialorphin (Gln–His–Asn–Pro–Arg)	C_26_H_42_N_12_O_8_	5.5^b^	650.3	651.4
II	Opiorphin (Gln–Arg–Phe–Ser–Arg)	C_29_H_48_N_12_O_8_	6.7^b^	692.3	693.2
III	[Ala^1^]sialorphin	C_24_H_39_N_11_O_7_	5.3^a^	593.3	594.8
IV	[Ala^2^]sialorphin	C_23_H_40_N_10_O_8_	5.2^a^	584.3	585.0
V	[Ala^3^]sialorphin	C_25_H_41_N_11_O_7_	5.7^b^	607.3	608.3
VI	[Ala^4^]sialorphin	C_24_H_40_N_12_O_8_	3.9^b^	624.3	625.7
VII	[Ala^5^]sialorphin	C_23_H_35_N_9_O_8_	6.1^b^	565.2	566.3
VIII	[His^2^]opiorphin	C_29_H_43_N_11_O_8_	7.8^b^	673.3	674.3
IX	[Ser^4^]sialorphin	C_24_H_40_N_12_O_9_	3.0^b^	640.3	641.4
X	[Arg^2^]sialorphin	C_26_H_47_N_13_O_8_	5.5^b^	669.3	670.2
XI	[Pro^4^]opiorphin	C_31_H_50_N_12_O_7_	9.33^b^	702.3	703.2

^a^A linear gradient from 2 to 60% B in 15 min

^b^A linear gradient from 2 to 40% B in 15 min, where [A] 0.1% TFA in water, [B] 0.1% TFA in acetonitrile, column Kromasil C8 (4.6 × 250 mm, pore size 100 Å, particle size 5 μm), flow rate 1.5 ml/min, *λ* = 214 nm

### Determination of Met-enkephalin degradation rates

The degradation studies were performed using the NEP enzyme extracted from porcine kidney, acquired from Merck (Warsaw, Poland). Solutions of Met-enkephalin, NEP and inhibitors were prepared by dissolving them in a Tris–HCl buffer (50 mM, pH 7.4). In investigated samples, the concentrations of Met-enkephalin, inhibitors and NEP were, respectively, 0.0413 mM, 0.156 mM and 5.687 nM. The reaction was initiated by addition of enzyme to a solution-containing Met-enkephalin and inhibitor. The samples consisting of the enzyme, Met-enkephalin and inhibitors were incubated over 0, 30, 60, 90, 120 min at 37 °C in a final volume of 300 μL. The reaction was stopped at a predetermined time by placing the tube on ice and acidifying with 30 μL of a 1 M aqueous HCl solution. The aliquots were centrifuged at 14,500 rpm for 20 min. The supernatants were filtered through Millipore Millex-GV syringe filters with 0.22 μm pores (Merck, Warsaw, Poland) and analysed by RP-HPLC on a Phenomenex Gemini-NX C18 column (5 μm, 4.6 mm × 150 mm) using a linear gradient of 3–50% of [B] for 8 min at a flow rate of 2.5 ml/min. Two solvent systems were used: 0.1% TFA in water [A] and 0.1% TFA in acetonitrile [B]. The rate constants of degradation (*k*) and degradation half-lives (*t*_1/2_) were calculated as described elsewhere (Kamysz et al. 2016; Perlikowska et al. 2012). All measurements were performed in triplicate.

### Molecular modelling

**Fig. 1:**
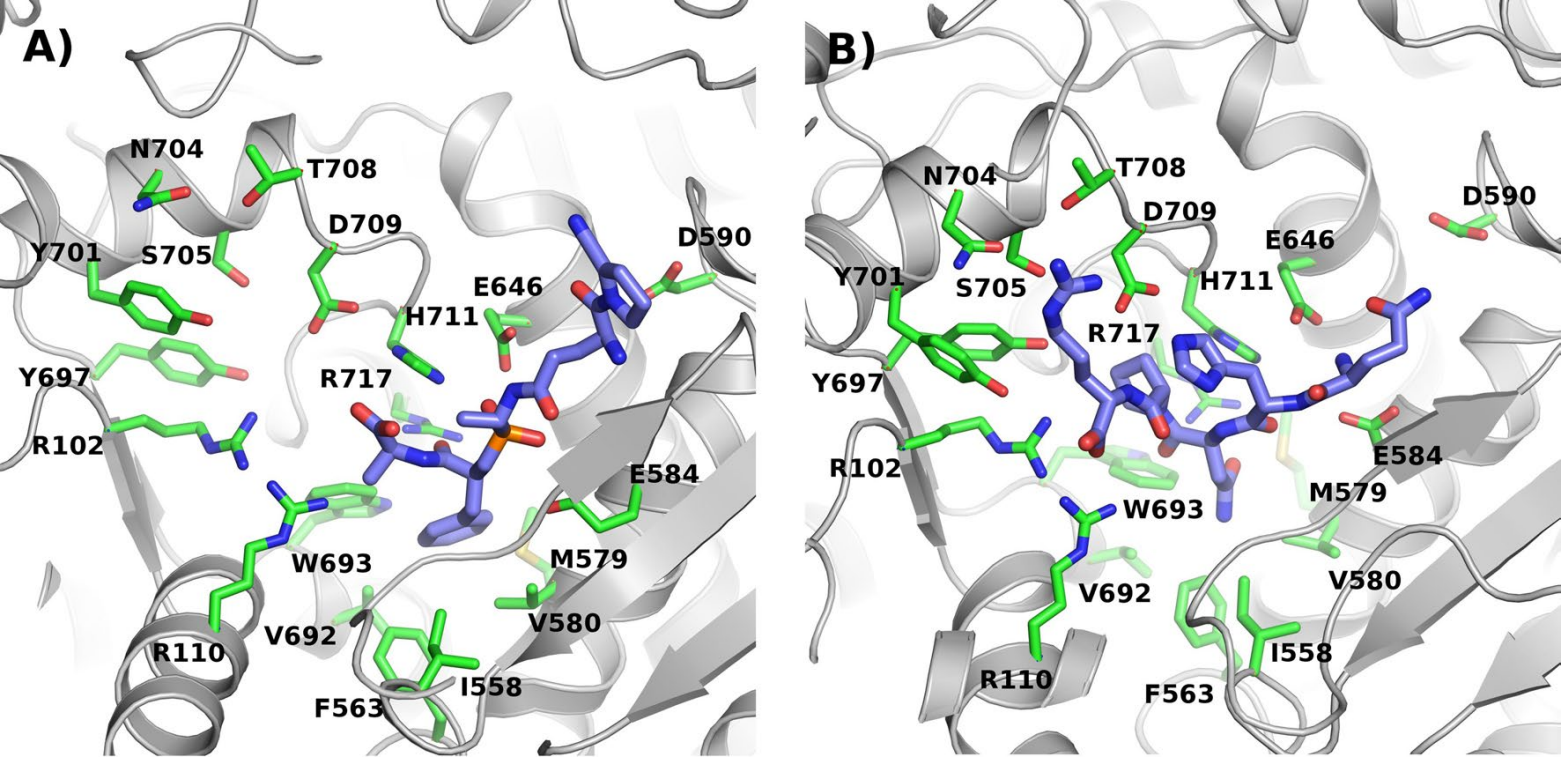
Visualization of the protein–peptide complexes. **a** The crystal structure of bifunctional NEP/DPP inhibitor, **b** theoretical structure of sialorphin bound to NEP

The sialorphin (Gln–His–Asn–Pro–Arg) complex was constructed similarly as reported elsewhere (Kamysz et al. 2016). In short, a human NEP structure (PDB ID: 2QPJ) with inhibitor was used as a template for modelling (Oefner et al. 2007). The inhibitor, as present in the 2QPJ structure was computer-mutated to sialorphin (Fig. 1a, b. The protein shape is marked in grey, ligand is marked in blue. The residues of protein interacting with peptide are shown in green). The phenyl ring of I20, located in the S1′ binding pocket, was substituted for asparagine residue since it is located in position (3). I20 is not a standard peptide, therefore, only the backbone trace was used for the proper location of the peptide. To obtain a low-energy structure, the model was optimised using minimization (500 cycles) and a short, low-temperature (2 ps, 50 K) molecular dynamics in repetitive cycles using AMBER v.12 (Pearlman et al. 1995; Case et al. 2005). This procedure was used to preserve the new model as much as possible in agreement with the experimental data. Subsequently, the model was analysed using the RasMol AB program (Pikora and Gieldon 2015).

### Statistics

Statistical analysis was performed using a Prism 5.0 (GraphPad Software Inc., CA, USA) software. The data are expressed as mean ± SEM. The Student’s *t* test followed by Newman–Keuls post hoc test was used for the analysis. *p* values < 0.05 were considered as statistically significant.

## Results and discussion

### Peptide synthesis and purification

Endogenous enkephalinase inhibitors opiorphin, sialorphin, alanine scan of sialorphin and four new hybrid peptides resulting from the combination of N- and C-terminal fragments of opiorphin and sialorphin were synthesised. The purity of peptides I–VIII after RP HPLC purification was higher than 98%. The identity of all peptides was confirmed by mass spectrometry MALDI-TOF and their found pseudomolecular ions values were in agreement with the expected theoretical values. The sequences and physicochemical characteristics of peptides I–VIII are shown in Table 1.

### Effect of alanine scan of sialorphin and its hybrids with opiorphin on degradation of Met-enkephalin by NEP

The in vitro studies of the inhibitory activity of opiorphin, sialorphin, alanine scan of sialorphin and their analogues against NEP were performed using a previously used method (Kamysz et al. 2016; Perlikowska et al. 2012). The degradation of Met-enkephalin was analysed by RP-HPLC as described in "Determination of Met-enkephalin degradation rates”.

Sialorphin and opiorphin are potent inhibitors of NEP as reported by us previously (Kamysz et al. 2016). Systematic Ala scanning of sialorphin (Table 2) showed that substitution of Gln^1^, Pro^4^ and Arg^5^ by Ala led to compounds with significantly reduced half-lives (*t*_1/2_) of Met-enkephalin. [Ala^2^]sialorphin displayed equivalent inhibitory potency for NEP as compared to that of sialorphin. [Ala^3^]sialorphin displayed a higher inhibitory potency for NEP as compared to that of sialorphin.

**Table 2:** Degradation rates (*k*) and half-lives (*t*_1/2_) of Met-enkephalin incubated with NEP alone and with inhibitors

Peptide	Inhibitor	1000 × *k* [1/min]	*t*_1/2_ [min]
	Without inhibitor	25.3 ± 1.0	27 ± 1
I	Sialorphin	8.8 ± 0.3***	78 ± 2***
II	Opiorphin	7.4 ± 0.2***	94 ± 2***
III	[Ala^1^]sialorphin	15.0 ± 0.3***	47 ± 1***
IV	[Ala^2^]sialorphin	9.0 ± 0.2***	74 ± 2***
V	[Ala^3^]sialorphin	6.0 ± 0.1***	109 ± 8***
VI	[Ala^4^]sialorphin	17.0 ± 0.8**	42 ± 2**
VII	[Ala^5^]sialorphin	13.0 ± 0.6***	52 ± 2***
VIII	[His^2^]opiorphin	5.9 ± 0.1***	116 ± 1***
IX	[Ser^4^]sialorphin	13.1 ± 0.4***	53 ± 1***
X	[Arg^2^]sialorphin	6.0 ± 0.1***	114 ± 2***
XI	[Pro^4^]opiorphin	5.0 ± 0.1***	139 ± 3***

Data are mean ± SEM

**p < 0.01, ***p < 0.001, compared to “without inhibitor”

The substitution of Pro^4^ for Ser^4^ in sialorphin (peptide IX—formed by fusion of three amino acids from N-terminal part of sialorphin and two amino acids from C-terminal part of opiorphin) led to a significant reduction of inhibitory activity. On the other hand, substitution of Ser^4^ for Pro^4^ in opiorphin (peptide XI—formed by fusion of three amino acids from N-terminal part of opiorphin and two amino acids from C-terminal part of sialorphin) extended the half-life of Met-enkephalin about five times in comparison to that of the sample without inhibitor. Peptides VIII and X exhibited a comparable activity and only slightly higher than sialorphin and opiorphin.

### Molecular modelling

Previous studies show that the N-terminal part of sialorphin has a strong influence on the NEP activity, since protection by the acetyl group depressed inhibition effect of the peptide (Kamysz et al. 2016). In our model (Fig. 1b), the amine group of the glutamine residue, located in position (1) of sialorphin, creates a salt bridge type interaction with Glu^584^ and Glu^646^ in NEP. This explains a depression of the inhibition effect following acetylation of the N-terminal group of sialorphin. In the NEP—(Gln–His–Asn–Pro-Arg) model (Fig. 1b), in contrast to the NEP—(Lys–Lys–Gln–Arg–Phe–Ser–Arg) one [as shown in our previous work (Kamysz et al. 2016)], in which the residue in position (1) was directed towards the cavity inside of the protein, here Gln in position (1) interacts with NEP. It seems that due to the steric effect, the inhibitor is taking different conformations with and without two additional residues (Lys–Lys) at the N-terminal part of the peptide (for comparison see reference Kamysz et al. 2016). Gln in position (1) is interacting with His^587^ and can form a weak hydrogen bond with Asp^590^. This result is partially confirmed by the Ala scan, since the mutation of glutamine to alanine depressed the inhibition effect (see peptide III).

His in position (2) is directed towards the cavity which is located in the protein interior. It seems to be in agreement with the experimental data, since we did not observe any significant change in the inhibition activity after the mutation of histidine to alanine (see peptide IV). However, if the residue is long enough to reach Asp^709^ and still the backbone is located in the protein binding pocket, we could observe increase of the inhibition effect (see peptide X). As it was pointed previously (Oefner et al. 2004; Misawa et al. 2011) and also as was found in the 2QPJ PDB structure, the residue located at the S1′ binding pocket should be hydrophobic.

The S1′ binding pocket is built from the following residues: Ile^558^, Phe^563^, Met^579^, Val^692^, Trp^693^, Val^580^ and Arg^717^. After the mutation of Asn in position (3) to alanine, most of those interactions disappeared (see peptide V). We may suspect that the observed increase of the inhibition effect is caused by removal of the hydrophilic residue from the highly hydrophobic pocket and better fit of the peptide to protein.

Pro in position (4) does not form any significant interactions with the protein (Arg^102^, Tyr^697^ and His^711^). The decrease of the inhibition activity of compounds VI and IX comparing to their parent peptide (sialorphin, I) can thus be explained by an enhanced conformational flexibility of the peptides. The increase of the inhibition activity of compound XI comparing to its parent compound (opiorphin, II) could be caused by decreased conformational flexibility of the peptide.

The mutation of Arg in position (5) to alanine (see peptide VII) weakened the inhibition effect of the peptide. Arg in position (5) is located in the vicinity of Ser^705^ and Thr^708^ that creates hydrogen bond interactions. The third residue located in this area is Asp^709^, however, we did not observe any salt bridge formed between Arg in position (5) and Asp^709^. We can thus assume that with this salt bridge the weakening of the inhibition effect (see peptides I and VII) would have been stronger. According to our model, the C-terminal part of the peptide can form a salt bridge interaction with Arg^110^ and/or Arg^102^. After protecting the C terminus by the amide group, the inhibitory effect was weakened as described previously (Kamysz et al. 2016).

## Conclusions

We performed an alanine scan of sialorphin to explore the role of its side chains in degradation of enkephalins by enkephalinases and showed that this compound has a critical structure which hardly tolerates substitutions in its primary sequence at positions 1, 4 and 5. The substitution of the amino acids Gln^1^, Pro^4^ and Arg^5^ of sialorphin for Ala reduces the half-life (*t*_1/2_) of Met-enkephalin in the presence of NEP. This result seems to be related to the influence of each residue on the peptide–enzyme interactions. With the molecular modelling techniques, we tried to obtain a reasonable explanation of the observed changes of the inhibition effect. The mutation of His^2^ to alanine in sialorphin was tolerated quite well. Substitution of Asn residue at position 3 in sialorphin for alanine residue (peptide V) as well replacement of Asn residue at position 3 in hybrid peptides IX and X by Phe residue led to compounds (respectively, peptides VIII and XI) with an enhanced inhibitory activity, owing to the introduction of a more hydrophobic amino acid residue, which has provided a better fit of the peptide to protein. This study provides important SAR information that may be applied in the design of new sialorphin analogues that could be useful pharmacological tools.

## Abbreviations

APN: Aminopeptidase N
ACE: Angiotensin-converting enzyme
DCM: Dichloromethane
DIC: *N*,*N*′-Diisopropylcarbodiimide
DIPEA: *N*,*N*-Diisopropylethylamine
DMF: *N,N*-Dimethylformamide
DMSO: Dimethyl sulfoxide
DPP III: Dipeptidyl peptidase III
Fmoc: 9-Fluorenylmethoxycarbonyl
HOBt: 1-Hydroxybenzotriazole
MALDI-TOF MS: Matrix-assisted laser desorption/ionization time-of-flight mass spectrometry
NEP: Neutral endopeptidase
Pbf: 2,2,4,6,7-Pentamethyldihydrobenzofuran-5-sulfonyl residue
RP-HPLC: Reversed-phase high-performance liquid chromatography
SAR: Structure–activity relationships
SPPS: Solid phase peptide synthesis
TFA: Trifluoroacetic acid
TIS: Triisopropylsilane
TNBS: 2,4,6-Trinitrobenzenesulfonic acid
Trt: Trityl
